# The Association between Healthy Beverage Index and Healthy and Unhealthy Obesity Phenotypes among Obese Women: A Cross-Sectional Study

**DOI:** 10.1155/2022/7753259

**Published:** 2022-12-29

**Authors:** Niloufar Rasaei, Dorsa Hosseininasab, Farideh Shiraseb, Fatemeh Gholami, Sahar Noori, Rasool Ghaffarian-Ensaf, Elnaz Daneshzad, Cain C. T. Clark, Khadijeh Mirzaei

**Affiliations:** ^1^Department of Community Nutrition, School of Nutritional Sciences and Dietetics, Tehran University of Medical Sciences (TUMS), Tehran, Iran; ^2^Department of Nutrition, Science and Research Branch, Islamic Azad University, Tehran, Iran; ^3^Non-Communicable Diseases Research Center, Alborz University of Medical Sciences, Karaj, Iran; ^4^Centre for Intelligent Healthcare, Coventry University, Coventry, CV1 5FB, UK

## Abstract

**Background:**

Metabolic phenotypes are new dimensions of obesity. Two important types of these phenotypes are metabolically healthy obesity (MHO) and metabolically unhealthy obesity (MUO). Studies showed that the components of the healthy beverage index (HBI) such as sugar-sweetened beverages (SSBs), milk, and fruit juices might have an association with MHO and MUO phenotypes.

**Methods:**

This cross-sectional study was performed on 210 women with the age range of 18–65 years and a body mass index (BMI) ≥25 kg/m^2^. The age range of the study population was the main inclusion criterion. Dietary intakes were assessed using a 147-item food frequency questionnaire (FFQ), as well as biochemistry and anthropometric parameters, in all participants. Metabolic health phenotypes were considered using the Karelis score, whilst HBI was evaluated based on 8 categories of beverages consumed. Analysis was carried out using binary logistic regression.

**Result:**

After controlling for a wide variety of confounding variables such as age, energy intake, BMI, education, physical activity, marriage, economic status, job, and supplementation, we found that the participants in the highest tertile of HBI had a lower risk of abnormal metabolic status than those in the lowest tertile (OR = 0.49; 95% CI: 0.07–3.21; *P* trend: 0.04), and it was not statistically significant, but we saw a significant trend.

**Conclusion:**

In conclusion, it seems that higher adherence to HBI can minimize the risk of metabolic abnormality, based on a significant trend.

## 1. Introduction

Obesity is a multifactorial and complex chronic disease that is defined by extra body weight according to height and is characterized by abnormal and excessive accumulations of fat in the body that cause adverse effects for health [1–3]. It has estimated that by 2025, 18% of men and 21% women will be considered as obese [4]. Obesity is associated with cardiovascular disease (CVD), disability, depression, type 2 diabetes, cancers, hypertension, dyslipidemia, and mortality [5, 6]. In 2015, there were about 4 million deaths worldwide due to obesity [7]. In Iran, studies showed that the burden of CVD will increase rapidly from 2005 to 2025, which in recent decades is leading to 46% of all deaths and 20%–23% of the burden of disease in Iran [8]. It has been reported (2015) an ascending trend in obesity above the age of 18, which was 21.7% [9].

People with obesity do not always have cardio-metabolic disease or other obesity complications [10, 11]. In this regard, there are some metabolic phenotypes in which two important types of these phenotypes are metabolically healthy obesity (MHO) and metabolically unhealthy obesity (MUO). MHO as a healthy obesity phenotype is seen in obese individuals who are protected against metabolic complications of obesity, and the risk of type 2 diabetes is lower than the MUO group [12–15]. MUO describes as lower level of insulin sensitivity, higher concentrations of systematic inflammation, high prevalence of hypertension, and hypertriglyceridemia [15, 16]. It is important to know that MHO can transited to MUO [17].

The healthy beverage index (HBI) was created to measure the quality of overall beverage intake [18]. This score included eight beverage categories such as water, coffee and tea, low fat milk, diet drinks, 100% fruit juices, alcohol, full fat milk, and sugar sweetened beverages (SSBs) [18, 19]. It has been revealed that healthy diets like Dietary Approach to Stop Hypertension (DASH) may prevent metabolic abnormalities in obese individuals[20]. The higher scores of HBI have been associated with better health outcomes, low risk of hypertension, and favorable lipid profile [18]. Finding from studies showed that the components of HBI such as SSBs [21–23], milk [24–26], and fruit juices [27] might have an association with obesity, and some studies that believe this component have no association with weight gain and adiposity [20, 28].

To our knowledge, no research has been conducted in this topic, and this is the first study that investigated the relationship between HBI and metabolically healthy and unhealthy obese phenotypes among obese women. Considering the association of HBI components with overweight and obesity, the importance of the type obesity, and its possibility to transition from MHO to MUO, we conducted this study to discover the association between HBI with MHO and MUO phenotypes among obese women.

## 2. Methods

### 2.1. Study Population

This cross-sectional study was conducted on obese women recruited between 2017 and 2019. Multistage simple random sampling was used. From all the health centers of the Tehran University of Medical Sciences (TUMS) of West and Central Tehran, using community-based sampling is based on comfort sampling. If women who were referred to Tehran health centers met the inclusion criteria, they were randomly selected to be included in the study [29]. Finally, 210 women were recruited. Women aged between 18 and 65 years who had a BMI ≥25 were eligible. The age range of the study population was the main inclusion criterion. Cancer and malignancies, kidney disease, liver disease, CVD, thyroid disease, menopause, smoking, diabetes types I and II, pregnancy, lactation, and any acute or chronic diseases, also taking weight-loss supplements, taking drugs to lower glucose, blood pressure, and lipid levels in plasma, and having a diet in the past year were the exclusion criteria. All the subjects signed a written informed consent form. The current study and the informed consent form were also accepted by the TUMS local ethics committee, with the ethics number IR.TUMS.MEDICINE.REC.1401.369.

### 2.2. Anthropometric Measurements

Body composition of all participants was determined by a bioelectrical impedance analyzer (BIA) (Inbody 770 Co., Seoul, Korea) following the manufacturer's guidelines [30]. We asked participants to remove metal utensils, shoes, and excess clothing. Body composition was measured after 12 hours of overnight fasting, between 8 and 9 am. Any uncommon physical activity for 72 h before the evaluation was avoided. BMI was measured with BIA, by dividing the body weight (kg) by the height (cm) squared. Body composition and weight, body fat mass (BFM), fat-free mass (FFM), fat mass index (FMI), and fat-free mass index (FFMI) body fat percentage were all checked in 15–20 seconds with BIA. Waist circumference (WC), at the narrowest girth, and hip circumference (HC), at the widest girth, were measured with a tape with an accuracy of 0.5 cm, according to standard anthropometric guidelines [31]. Waist-to-hip ratio (WHR) was measured by dividing the waist circumference by the hip circumference.

### 2.3. Dietary Assessment and HBI Definition

Dietary intake of all participants during the last year was assessed with a reliable and validated semiquantitative 147-item food frequency questionnaire (FFQ) [32]. For converting the dietary intake of participants into grams and milliliters and to assess the dietary intake data, we used the Nutritionist 4 (Hearst Corporation, San Bruno, CA) food analyzer. The Nutritionist 4 program is used to compute total energy, macronutrients, and micronutrients (Hearst Corporation, San Bruno, CA) [33].

Duffey and Davy [18] established a method for determining the HBI. Water, 100 percent fruit juice, skimmed milk, unsweetened coffee/tea, diet drinks (including zero-calorically sweetened coffee/tea and other beverages with artificial sweeteners), alcohol (including wine, beer, and liquor), whole milk, and SSBs (including sweetened coffee/tea, soda, and fruit drinks) were the eight categories of beverages consumed. The final HBI score ranges from 0 to 100, with higher numbers indicating better compliance with beverage standards and a healthy beverage consumption pattern [18]. In this study, the maximum final HBI score was 90 because diet drinks and alcohol (with a score ranging from 0 to 5) were not consumed by our objective group. Liquids consumed as part of a meal (e.g., soup) were omitted because the purpose of this study was to assess adherence to healthy beverage intake guidelines rather than total liquid intake. Our subjects did not consume these items, resulting in a maximum final HBI score of 90. This study aimed to monitor how well people adhered to the rules.

### 2.4. Metabolic Health Phenotypes

To evaluate metabolic health, the Karelis criterion was used: triglycerides (TG) ≤1.7 mmol/L or taking lipid-lowering drugs, high-density lipoprotein (HDL-C) ≥ 1.3 mmol/L, low-density lipoprotein (LDL-C) ≤ 2.6 mmol/L, insulin resistance homeostatic model assessment (HOMA) ≤2.7, and CRP ≤3.0 mg/L [34]. Individuals were then classified into two groups: MHO and MUO phenotypes [15, 35]. Unhealthy phenotype occurs when at least three of the above components are met.

### 2.5. Assessment of IPAQ

We used an international physical activity questionnaire-short form (IPAQ) to determine the physical activity level (PA) of participants. Over the past year, IPAQ contained the duration and frequency of routine activities in each week of daily life. Individuals PA levels were measured in metabolic equivalent hours per week (METs-h/week) [36].

### 2.6. Assessment of Other Variables

Blood pressure was measured by a trained physician using a standard sphygmomanometer (Omron, Germany, Europe). Demographic questionnaires collect data on age, level of education (illiterate, diploma, bachelor, and higher), marital status (single and married), specific diet, medical history, medication, and supplementation which was completed by trained nutritionists.

### 2.7. Biochemical Assessment

After a night fasting, venous blood was collected between 8:00 and 10:00 in the morning. All blood samples were centrifuged and then were kept at −80°C. All the samples were evaluated using a single assay procedure. The fasting blood sugar (FBS) was measured using the GOD/PAP (glucose oxidase phenol 4-aminoantipyrine peroxidase) method. The enzyme-linked immunosorbent assay (ELISA) method was used to determine serum insulin concentrations. The HOMA-IR was calculated through this equation: [fasting plasma glucose (mmol/l)  ×  fasting plasma insulin (mIU/l)]/22.5 [37]. The enzymatic endpoint glycerol-3-phosphate oxidase phenol 4-aminoantipyrine peroxidase (GPOPAP) was utilized to evaluate total cholesterol (TC) levels and TG. HDL and LDL cholesterol were assessed using a direct enzymatic clearance test. For quantifying serum hs-CRP levels, an immunoturbidimetric test using a Pars Azmoon kit was used (Pars Azmoon Inc. Tehran, Iran). All samples were evaluated in The Nutrition and Biochemistry Laboratory of the TUMS School of Nutritional and Dietetics using methodologies which has been established.

### 2.8. Statistical Analysis

SPSS software (version 23, SPSS Inc., Chicago, IL, USA) was used to conduct statistical analysis, and *P* < 0.05 was set as statistically significant. Normality of the data was checked by the Kolmogorov–Smirnov test. Categorical factors (job, educational status, income, supplement use, and marriage) were presented as number and percent across HBI groups, and it was performed using the chi-square test. The analysis of variance (ANOVA) test was used to compare the continuous variables between tertiles of the HBI. Estimating energy-adjusted participant's dietary intakes between tertiles of a HBI was done by the analysis of covariance (ANCOVA) test. The binary logistic regression test was performed for assessing the association between MHO and MUHO and HBI in three different models: crude model; model 1 was adjusted for energy intake, age, and BMI; and model 2 was adjusted for education status plus model 1.

## 3. Results

The statistical analysis comprised 210 women in all. The means and standard deviation (SD) of the HBI in this study were 65.12 ± 4.40. The mean (SD) of age, weight, BMI, WC, and body fat mass of individuals were 36.22 ± 8.56 years, 80.42 ± 12.00 kg, 30.79 ± 4.17 kg/m^2^, 95.37 ± 15.78 cm, and 33.42 ± 8.11 kg, respectively.

Socioeconomic status, such as marriage, occupation, education, and economic standing, were also examined in this cross-sectional study. The findings revealed that 202 (96.2%) of the participants were employed and 153 (72.9%) of the participants were married. The majority of participants had received a diploma (76 (36.2%)) or a bachelor's degree or above (101 (48.1%)). In this survey, 54 (25.7%) of the participants had an excellent financial situation.

Table 1 shows the frequency of metabolic status based on Karelis criteria. Seventy-one percent of those who took part in the study had metabolic problems.

### 3.1. Difference in Mean of Body Composition, Blood Pressure, and Blood Sample across HBI

Table 2 shows the characteristics of the participants with respect to the different categories of the HBI. In the adjusted model, there was a significant difference across HBI for HDL (*P*=0.006) and LDL (*P*=0.009). After adjusting for confounding factors (BMI, age, physical activity, and energy intake), the results demonstrated a borderline significant difference across the HBI for diastolic blood pressure (*P*=0.07) and triglyceride (*P*=0.06). There were no significant differences between the HBI and other factors.

### 3.2. Comparison of Daily Nutrients Intake in Participants across HBI

Table 3 shows the nutritional and food group intake of subjects across tertiles of HBI. After adjusting for energy intake, participants in the highest category of HBI had a higher intake of potassium (*P*=0.02), vitamin B6 (*P*=0.005), biotin (*P*=0.01), whole grains (*P*=0.004), and fruits (*P*=0.02), and a lower intake of monounsaturated fatty acid (MUFA). There was also a marginally significant variation in copper intake (*P*=0.06). Vitamin E intake was higher in the lowest tertile of HBI participants (*P*=0.01).

### 3.3. Association of the HBI with Metabolic Status

Table 4 shows the risk of having abnormal metabolic status by tertiles of HBI. After controlling for potential covariates (age, BMI, energy intake, education, marital status, physical activity, job, economic status, and supplementation) in the binary logistic regression analysis (model 3), participants in the top tertile of HBI were less likely to have abnormal metabolic status than those in the bottom tertile (OR: 0.49; 95% CI: 0.07–3.21; *P*=0.46), and it was not statistically significant, but a significant trend was observed (*P* trend: 0.04). So, the findings of this study revealed that greater adherence to a healthy beverage intake can minimize the risk of metabolic abnormality (*P* trend: 0.04).

## 4. Discussion

To our knowledge, this is the first study designed to assess the relationship between HBI and obesity metabolic phenotypes in obese Iranian women.

It seems that participants who had higher HBI scores were less likely to have abnormal metabolic status although we did not see any significant association (*P*=0.46). However, the findings of this study revealed that greater adherence to HBI may minimize the risk of metabolic abnormality as we saw a reasonable and significant trend (*P* trend: 0.04). This trend may be in line with other studies that showed adherence to a healthy diet such as DASH diet that may reduce the odds of MUO [38]. It has been reported that MHO can be transited to MUO during the time, so the risk of abnormalities such CVD can increase [17]. A cross-sectional study in 2017 showed that healthy beverages intake has a relationship with a lower risk of metabolic abnormalities and abdominal obesity [39]. In the current study, individuals in the third tertile of HBI had lower levels of TG, DBP, and LDL and higher levels of HDL. Duffey and Davy indicated that higher HBI scores were associated with a lower risk of hypertension and also lower levels of C-reactive protein, insulin, FBS, and cholesterol. They also expressed that HBI might be an invaluable tool to evaluate overall beverage intake quality in adults [18]. The conflicting results may be related to the difference in the characteristics of the study samples. Another thing that might influence our results is the fact that most of our study participants were metabolically unhealthy (71%). As we mentioned, Duffey and Davy saw a negative association between HBI scores and hs-CRP. One study in 2014 reported that obesity was associated with chronic inflammation in obese subjects [40]. Adipose tissue produces various pro-inflammatory and anti-inflammatory factors, including the adipokines adiponectin, leptin, and resistin, as well as chemokines and cytokines, such as IL-6, TNF-*α*, and MCP-1 [41, 42]. Thus, monitoring and controlling obesity metabolic phenotypes in patients can reduce future consequences such as chronic inflammation.

In the present study, consumption of potassium, vitamin B6, whole grains, and fruits was positively associated with a tertile of the HBI scores. In line with our study, a cross-sectional study in Korea showed that subjects who adhered to the healthy beverage pattern had a higher intake of whole grains, noodles, flour, bread, fruits, and healthy beverages such as unsweetened tea, dairy products, and fruit and vegetable juices [39]. Park et al. in a randomized clinical trial (RCT) study reported that cabbage-apple juice might have beneficial effects on lipid metabolism dysfunction and obesity-related abnormalities in the rat model [43]. Additionally, a review article suggested that an increased water intake has a relationship with body weight loss through decreased feeding and increased lipolysis [44]. Another study in 2022 displayed that participants who consumed fruits more often (6-7 days per week compared to 0–2 days per week) had lower risks of high triglycerides, dyslipidemia, and hyperlipidemia [45]. Fruit and vegetable juices can be vital sources of vitamins A, C, E, K, and B-6, thiamin, niacin, folate, and choline, as well as potassium, iron, manganese, and fiber [46]. Moreover, phytonutrients such as flavonoids and carotenoids, contained in fruit and vegetable juices, have numerous health benefits [47].

In our study, DBP, TG, and LDL levels were negatively associated, whereas HDL levels were positively associated with a tertile of the HBI scores. In accordance with our findings, an RCT study found that consumption of green tea as a part of healthy beverages caused a moderate but significant decrease in LDL cholesterol levels compared to the control group. They also assessed the antioxidative status and suggest that drinking green tea could protect humans against oxidative damage [48]. Similarly, a meta-analysis assessing black tea consumption on blood pressure expressed that 4-5 cups of black tea decreased systolic and diastolic blood pressure by 1.8 and 1.3 mmHg, respectively [49]. Mashhadi et al. evaluated the association between beverage consumption patterns and lipid profile in shift workers and found that shift workers had lower intakes of water and higher intakes of high-sugar beverages and their TG levels were also higher than day workers [50].

Finally, we observed a significant decreasing trend (p trend: 0.04) in unhealthy metabolic status with an increase in HBI scores although it was not statistically significant (*P* value: 0.46). Our study was performed only on obese women. The study may show different results with a different study populations, and since this is the first study to identify the association between HBI and obesity metabolic phenotypes in Iranians, our findings cannot be directly compared with the results from the previous studies.

There are different mechanisms through which healthy beverages may be associated with obesity. Physiologically, augmented healthy beverage intake, especially water, leads to an increase in blood volume which is followed by an increase in right atrium pressure. As a result, atrial natriuretic peptide (ANP), one of the first natriuretic peptides discovered, would be released [51]. This family of cardiac natriuretic peptides activates uncoupling protein 1 (UCP1) that enhances metabolism of fat and results in body weight loss [52, 53]. Another feasible mechanism is that increased water intake (as a part of healthy beverages) drives thermogenesis [54–57] that also would results to a reduction in weight gain.

On the other hand, increased consumption of SSBs is positively related to the obesity risks and metabolic syndrome (MetS) components. Exclusively, the levels of insulin and glucose in blood increased rapidly after ingestion of SSBs [58]. This is because of the existence of high-fructose corn syrup or sucrose, which are commonly used to flavor SSBs and results in a high dietary glycemic load. Since these substances leads to a low level of satiety, people have a tendency to consume greater calories, resulting in obesity and weight gain [59]. In addition, it has been expressed that the high contents of sugar in SSBs could instantly result in glucose intolerance or insulin resistance, which in turn augmented the risk of dyslipidemia and elevated FBG [60]. Previous studies showed that SSBs which have a relationship with the increased risk of obesity and obesity-related diseases were an important part of a specific dietary pattern [61, 62].

This study has several strengths and limitations. First, this is the first study that investigated the association between HBI and metabolic phenotype obesity in obese Iranian women. Second, evaluating the dietary intakes by using a locally validated questionnaire (FFQ) was done by an experienced dietitian to decrease measurement errors. Nonetheless, there are a few limitations to be considered. First, in cross-sectional studies, causal associations could not be suggested. Second, because of misremembering the data and misclassification errors, small errors might exist in the dietary assessment. As a third limitation, the results of our study only apply to women, and it is inapplicable to men. Finally, larger sample sizes could detect smallest difference between groups.

## 5. Conclusion

In conclusion, it seems that higher adherence to HBI can minimize the risk of metabolic abnormality, and this association was not statistically significant, but a significant trend was observed. Further studies are needed to draw more definitive conclusions and process scientific guidelines for achieving healthy beverage consumption.

## Figures and Tables

**Table 1:** Frequency of metabolic status based on Karelis criteria.

Variables	Karelis criteria	Number (%)
TG (mg/dl)	<1.7	150 (71.4%)
	>1.7	49 (23.3%)

HDL_C (mg/dl)	>1.3	61 (29%)
	<1.3	138 (65.7%)

LDL_C (mg/dl)	<2.6	130 (61.9%)
	>2.6	69 (32.9%)

Hs-CRP (mg/l)	<3	114 (54.3%)
	>3	85 (40.5%)

HOMA-IR	<2.7	59 (28.1%)
	>2.7	137 (65.2%)

Metabolic status	MH	47 (22.4%)
	MUH	149 (71%)

HDL: high-density lipoprotein; hs-CRP: high-sensitivity C- reactive protein; HOMA: homeostatic model assessment; LDL: low-density lipoprotein; TG: triglyceride; MH: metabolic healthy; MUH: metabolic unhealthy.

**Table 2:** Mean and SD of anthropometric body composition, blood parameters, and blood pressure according to tertiles of the healthy beverage index.

Variables^†^	HBI				
	T1 (*n* = 75)	T2 (*n* = 80)	T3 (*n* = 55)	*P* value	*P* value^*∗*^
Age (years)	36.25 ± 8.43	36.18 ± 9.05	35.95 ± 8.04	0.98	0.09
Body weight (kg)	80.83 ± 10.92	78.89 ± 11.93	81.63 ± 9.78	0.37	0.12
Height (cm)	161.65 ± 5.50	161.09 ± 5.46	162.40 ± 6.15	0.47	0.20
BMI (kg/m^2^)	30.88 ± 3.57	30.46 ± 3.95	30.76 ± 2.99	0.76	0.62
IPAQ (MET min-week)	1018.55 ± 2014.21	1134.36 ± 1243.87	1884.14 ± 3614.18	0.14	0.58

*Body composition*					
WC (cm)	93.74 ± 16.83	93.89 ± 9.85	99.31 ± 21.63	0.17	0.09
HC (cm)	113.90 ± 9.24	112.42 ± 8.14	114.32 ± 6.62	0.44	0.60
WHR	0.92 ± 0.04	0.93 ± 0.05	0.94 ± 0.04	0.08	0.12
BFM (kg)	33.50 ± 7.55	32.77 ± 7.70	33.66 ± 5.76	0.76	0.53
BF (%)	41.14 ± 5.29	40.91 ± 4.73	40.77 ± 5.67	0.93	0.94
FFM	47.33 ± 5.80	46.34 ± 5.89	47.23 ± 5.03	0.53	0.11
FMI	12.88 ± 2.85	12.63 ± 2.88	13.01 ± 2.57	0.75	0.76
FFMI	18.07 ± 1.50	17.82 ± 1.50	20.76 ± 19.19	0.22	0.38

*Blood pressure*					
SBP (mmHg)	113.92 ± 11.91	111.73 ± 14.45	111.06 ± 12.63	0.47	0.12
DBP (mmHg)	80.51 ± 8.62	78.33 ± 12.04	76.53 ± 9.01	0.12	**0.07**

*Blood sample*					
FBS (mmol/L)	4.79 ± 0.57	4.74 ± 0.50	4.95 ± 0.54	0.09	0.49
Total cholesterol (mmol/L)	4.63 ± 0.79	4.74 ± 1.03	5.15 ± 1.00	**0.01**	0.10
TG (mmol/L)	1.56 ± 0.86	1.36 ± 0.74	1.35 ± 0.88	0.27	**0.06**
HDL (mmol/L)	1.11 ± 0.20	1.23 ± 0.27	1.12 ± 0.34	**0.01**	**0.006**
LDL (mmol/L)	2.57 ± 0.53	2.53 ± 0.68	2.10 ± 0.61	**<0.001**	**0.009**
hs-CRP (mg/L)	5.17 ± 5.15	4.59 ± 4.76	4.08 ± 4.20	0.49	0.97
HOMA index	3.31 ± 1.35	3.47 ± 1.40	3.75 ± 1.11	0.22	0.76
Insulin (mIU/ml)	1.23 ± 0.22	1.22 ± 0.26	1.23 ± 0.22	0.92	0.87

*Education category*					
Illiterate	0 (0.0)	2 (66.7)	1 (33.3)	0.23	
Under diploma	4 (16.0)	13 (52.0)	8 (32.0)		
Diploma	25 (35.7)	29 (41.4)	16 (22.9)		
Bachelor and higher	37 (42.0)	29 (33.0)	22 (25.0)		

*Marital status*					
Single	18 (36.7)	20 (40.8)	11 (22.4)	0.86	
Married	48 (35.0)	53 (38.7)	36 (26.3)		

*Supplement intake*					
Yes	38 (49.4)	31 (40.3)	8 (10.4)	0.54	
No	25 (43.9)	28 (49.1)	4 (7.0)		

*Job category*					
Employed	67 (37.0)	70 (38.7)	44 (24.3)	0.21	
Unemployed	0 (0.0)	0 (0.0)	1 (100.0)		

*Economic status*					
Poor	25 (51.0)	13 (26.5)	11 (22.4)	0.18	
Moderate	25 (34.2)	33 (45.2)	15 (20.5)		
Good	15 (31.9)	19 (40.4)	13 (27.7)		

SD: standard deviation; HBI: healthy beverage index; BMI: body mass index; WC: waist circumference; HC: hip circumference; WHR: waist height ratio; FFM: fat-free mass; FMI: fat mass index; FFMI: fat-free mass index; BFM: body fat mass; BF: body fat; SBP: systolic blood pressure; DBP: diastolic blood pressure; FBS: fasting blood sugar; TG: triglyceride; LDL: low-density lipoprotein; HDL: high-density lipoprotein; hs-CRP: high-sensitivityC-reactive protein; HOMA: homeostatic model assessment. ^†^Calculated by analysis of variance (ANOVA). Analysis of covariance (*P* value^*∗*^) was performed to adjust potential confounding factors (age, BMI, energy intake, physical activity). BMI considers as the collinear variable for anthropometrics and body composition variables. Values are represented as means (SD). Categorical variables: *N* (%). *P* values <0.05 were considered as significant. The bold values are the values <0.05 and considered significant, or the values that are considered marginally significant.

**Table 3:** Nutrient intake according to tertiles of healthy beverage index.

Variables	HBI				
	T1 (*n* = 75)	T2 (*n* = 80)	T3 (*n* = 55)	*P* value	*P* value^*∗*^
*Dietary intakes*					
Energy (kcal/d)	2511.26 ± 690.39	2645.40 ± 769.13	2569.56 ± 782.68	0.58	
Total fat (g/d)	91.80 ± 32.56	96.54 ± 31.05	86.56 ± 31.26	0.26	0.11
Cholesterol (mg/d)	236.98 ± 102.11	271.73 ± 117.47	252.75 ± 81.39	0.15	0.36
SFA (mg/d)	26.47 ± 10.28	29.87 ± 12.50	25.61 ± 10.15	0.09	0.09
MUFA (g/d)	31.70 ± 12.61	31.03 ± 10.41	27.94 ± 9.67	0.19	**0.02**
PUFA (g/d)	20.46 ± 9.92	19.72 ± 7.71	17.73 ± 7.88	0.26	0.12
Linolenic acid (g/d)	1.05 ± 0.61	1.48 ± 0.67	1.11 ± 0.53	**<0.001**	**<0.001**
Linoleic acid (g/d)	18.05 ± 9.53	16.60 ± 7.10	15.80 ± 7.38	0.19	0.08
EPA (mg/d)	0.03 ± 0.04	0.03 ± 0.03	0.03 ± 0.04	0.94	0.73
DHA (mg/d)	0.11 ± 0.12	0.11 ± 0.11	0.11 ± 0.12	0.95	0.72
TFA (mg/d)	0.0008 ± 0.001	0.001 ± 0.002	0.001 ± 0.004	0.54	0.58
Sodium (mg/d)	42.03 ± 1393.40	4304.40 ± 1303.65	4003.29 ± 1370.35	0.51	0.37
Potassium (mg/d)	4106.18 ± 1582.21	4412.53 ± 1452.27	4718.50 ± 1638.44	0.12	**0.02**
Vitamin A (RAE)	723.07 ± 364.79	849.82 ± 435.02	849.82 ± 486.89	0.15	0.28
Β-carotene (mg/d)	4718.43 ± 2765.62	5782.88 ± 3786.84	6089.74 ± 5000.33	0.12	0.18
Vitamin D (*μ*g/d)	2.01 ± 1.59	1.87 ± 1.37	2.00 ± 1.32	0.84	0.67
Vitamin E (mg/d)	19.16 ± 10.81	15.03 ± 6.18	15.15 ± 7.95	**0.01**	**0.002**
Thiamin (*μ*g/d)	2.01 ± 0.63	2.08 ± 0.65	2.01 ± 0.68	0.82	0.68
Riboflavin (mg/d)	2.15 ± 0.90	2.25 ± 0.75	2.27 ± 0.90	0.70	0.77
Niacin (mg/d)	23.56 ± 7.31	26.17 ± 10.09	25.32 ± 10.52	0.25	0.45
Vitamin B6 (*μ*g/d)	2.00 ± 0.64	2.23 ± 0.76	2.32 ± 0.74	**0.04**	**0.005**
Folate (*μ*g/d)	584.85 ± 181.50)	610.61 ± 171.28	621.80 ± 182.40	0.52	0.41
Vitamin B12 (*μ*g/d)	4.16 ± 1.96	4.79 ± 2.51	4.45 ± 2.71	0.30	0.55
Biotin	36.97 ± 15.01	38.50 ± 13.23	45.48 ± 26.75	**0.04**	**0.01**
Pantothenic acid	6.32 ± 2.21	6.39 ± 1.89	6.93 ± 3.81	0.44	0.15
Vitamin K (*μ*g/d)	185.00 ± 109.43	249.58 ± 217.43	270.51 ± 350.89	0.11	0.15
Phosphorus (mg/d)	1596.40 ± 504.78	1695.12 ± 546.73	1617.85 ± 545.03	0.53	0.86
Vitamin C *μ*mol/L)	183.10 ± 165.48	191.32 ± 103.28	225.61 ± 117.66	0.24	0.15
Calcium (mg/d)	1127.46 ± 416.04)	1197.17 ± 420.44	1138.66 ± 424.91	0.60	0.86
Iron (mg/d)	18.04 ± 6.06	19.15 ± 5.91	18.86 ± 6.67	0.56	0.82
Magnesium (mg/d)	449.03 ± 150.26	472.64 ± 147.58	480.39 ± 162.81	0.51	0.41
Zinc (mg/d)	12.81 ± 4.45	13.39 ± 4.20	13.11 ± 4.57	0.74	0.97
Copper (mg/d)	1.89 ± 0.63	2.04 ± 0.61	2.15 ± 1.05	0.18	**0.06**
Manganese (mg/d)	7.09 ± 2.45	7.33 ± 2.80	7.21 ± 3.84	0.89	0.98
Selenium (mg/d)	120.48 ± 38.00	121.01 ± 44.21	115.76 ± 47.75	0.79	0.35
Chromium (mg/d)	0.12 ± 0.08	0.11 ± 0.09	0.09 ± 0.08	0.26	0.15
Total fiber (g/d)	42.94 ± 19.56	45.41 ± 16.91	45.92 ± 18.48	0.63	0.77
Caffeine (mg/d)	139.65 ± 103.40	172.09 ± 126.52	172.88 ± 296.41	0.49	0.53

*Food groups*					
Whole grains (g/d)	6.88 ± 7.93	7.16 ± 11.21	13.67 ± 15.02	**0.004**	**0.004**
Fruits (g/d)	488.09 ± 379.20	510.24 ± 303.14	639.12 ± 378.77	**0.07**	**0.02**
Vegetables (g/d)	423.42 ± 264.40	454.20 ± 235.00	531.20 ± 341.35	0.12	0.12
Nuts (g/d)	13.4423 ± 17.57	17.31 ± 16.32	16.60 ± 20.71	0.42	0.48
Legumes (g/d)	56.26 ± 46.30	55.90 ± 43.10	56.09 ± 44.65	0.99	0.99
Sweets. desserts (g/d)	35.07 ± 29.46	77.14 ± 233.31	44.20 ± 36.24	0.22	0.31
Refined grains (g/d)	463.80 ± 239.66	452.18 ± 202.81	477.23 ± 316.72	0.87	0.48
Dairy (ml/d)	384.97 ± 242.53	380.32 ± 212.35	408.13 ± 315.66	0.84	0.65
Eggs (g/d)	20.54 ± 13.41	21.96 ± 13.57	24.97 ± 15.74	0.26	0.32
White meat (g/d)	39.95 ± 29.53	53.93 ± 55.54	44.15 ± 34.36	0.15	0.29
Red meat (g/d)	18.99 ± 15.26	27.36 ± 23.35	23.51 ± 21.59	**0.05**	0.10

DHA: docosahexaenoic acid, EPA: eicosapentaenoic acid, MUFA; monounsaturated fatty acid, PUFA: polyunsaturated fatty acid, SFA: saturated fatty acid, TFA: trans fatty acid. Values are represented as means SDANCOVA (*P* value^*∗*^) was performed to adjusted potential confounding factors (energy intake). *P* values <0.05 were considered as significant. The bold values are the values <0.05 and considered significant, or the values that are considered marginally significant.

**Table 4:** Odds ratio and 95% confidence interval for the association between tertiles of healthy beverage index and metabolic status.

	HBI					
	T1^*∗*^	T2	*P* value	T3	*P* value	*P* trend
Crude	1.00	0.94 (0.41–2.11)	0.88	1.28 (0.49–3.37)	0.60	0.64
Model 1	1.00	0.79 (0.30–2.08)	0.64	1.01 (0.28–3.65)	0.97	0.93
Model 2	1.00	0.62 (0.21–1.83)	0.39	0.90 (0.22–3.65)	0.89	0.59
Model 3	1:00	0.60 (0.21–1.75)	0.35	0.49 (0.07–3.21)	0.46	**0.04**

Based on binary regression model in crude and adjustment models. ^*∗*^Tertile 1 considered as the reference group. Model 1: adjusted for age, BMI, energy intake, and physical activity. Model 2: adjusted for age, BMI, energy intake, physical activity, education, marital status, economic status, and job. Model 3: adjusted for model2 + supplementation. The bold values are the values <0.05 and considered significant.

## Data Availability

The data are available upon request to the corresponding author.

## Abbreviations

BF: Body fat
BMI: Body mass index
CVD: Cardiovascular disease
FBS: The fasting blood sugar
FFMI: Fat-free mass index
FFM: Fat-free mass
FMI: Fat mass index
FFQ: Food frequency questionnaire
HBI: Healthy beverage index
HDL: High-density lipoprotein
HOMA-IR: Insulin resistance homeostatic model assessment
IL-1: Interleukin-1
IPAQ: International physical activity questionnaire-short form
LDL: Low-density lipoprotein
MHO: Metabolically healthy obesity
MUO: Metabolically unhealthy obesity
SSBs: Sugar-sweetened beverages
TC: Total cholesterol levels
TG: Triglyceride
TNF-*α*: Tumor necrosis
WC: Waist circumference
WHR: Waist-to-hip ratio, facto.
